# Metaplasticity in swallowing system via cross-modal neurostimulation: A randomized crossover trial with magnetoencephalography

**DOI:** 10.1016/j.neurot.2026.e00835

**Published:** 2026-01-21

**Authors:** Ivy Cheng, Anne Jung, Bendix Labeit, Rainer Dziewas, Andreas Wollbrink, Joachim Gross, Paul Muhle, Sonja Suntrup-Krueger

**Affiliations:** a Academic Unit of Human Communication, Learning, and Development, Faculty of Education, University of Hong Kong, Hong Kong; b Centre for Gastrointestinal Sciences, Faculty of Biology, Medicine and Health, University of Manchester, United Kingdom; c Institute for Biomagnetism and Biosignal Analysis, University of Muenster, Germany; d Department of Neurology, University Hospital Muenster, Germany; e Department of Neurology, Medical Faculty and University Hospital Duesseldorf, Heinrich Heine University, Duesseldorf, Germany; f Department of Neurology, Osnabrueck Hospital, Germany

**Keywords:** Brain stimulation, Magnetoencephalography, Metaplasticity, Pharynx, Swallowing

## Abstract

The response to neurostimulation can be modulated based on the state of neural network activation prior to stimulation, a mechanism termed metaplasticity. In the swallowing system, preconditioning the pharyngeal motor cortex with non-invasive brain stimulation (NIBS) can induce metaplasticity. However, the effects of cross-modal neurostimulation, i.e. combined peripheral (pharyngeal electrical stimulation; PES) and central (transcranial direct current stimulation; tDCS) approaches, remain unknown. This study investigated the effects of PES preconditioned by tDCS on cortical activation of swallow-related network and swallowing behaviour. Twenty-one healthy volunteers (8 males, 13 females; mean age = 27.0 ± 4.8 years) participated in the study. They received, in randomized order across three separate visits, three experimental conditions in which PES (5Hz, 10 min) was preconditioned by (1) anodal tDCS (1 mA, 20 min), (2) cathodal tDCS (1.5 mA, 10 min), and (3) sham tDCS. Cortical activation of swallow-related network during “volitional” and “challenged” swallow tasks was measured using magnetoencephalography (MEG) at baseline and immediately post-intervention. Swallowing behaviour was assessed by submental electromyographic (EMG) measurements and timed water swallow test. Significant bilateral enhancement of cortical activation of swallow-related network during challenged swallow task was observed in the theta frequency following cathodal preconditioning (*p* = 0.042) and PES alone (*p* < 0.001). By contrast, anodal preconditioning significantly reduced swallow-related network activation in the alpha frequency (*p* = 0.037). There were no significant changes in swallowing behaviour across conditions. This is the first evidence of metaplasticity induced by cross-modal neurostimulation in the swallowing system. Future studies may focus on its clinical application in patients with neurogenic dysphagia.

## Introduction

Swallowing is a highly complex process mediated by central and peripheral neural networks, and damage to these following neurological conditions such as stroke often results in dysphagia [1,2]. Patients with dysphagia may develop life-threatening complications such as malnutrition, dehydration, or aspiration pneumonia [3,4].

Pharyngeal electrical stimulation (PES) and transcranial direct current stimulation (tDCS) are neurostimulation techniques that promote structural and functional neuroplasticity of the swallowing neural network which facilitates functional recovery from neurogenic dysphagia [5,6]. Compensatory neural adaptations also occur in patients with Parkinson's disease and bulbospinal muscular atrophy who retained functional swallowing [7,8]. PES drives long-term neuroplastic changes by delivering electric current to the pharyngeal sensory afferents [[9], [10], [11]]. Studies have reported its modulatory effects in cortical activation sensorimotor swallowing network [12,13] and neuropeptide substance P level [14]. By contrast, tDCS modulates brain activities with electric current delivered through surface scalp electrodes [15,16]. Anodal and cathodal tDCS increases and decreases cortical excitability of the pharyngeal motor cortex respectively [17]. Enhanced swallow-related cortical activation induced by anodal tDCS is associated with improved performance in complex swallow tasks [18]. Clinically, tDCS and especially PES have shown benefits for patients with post-stroke dysphagia in randomized controlled phase II and III trials [[19], [20], [21], [22], [23], [24]]. Most evidence for the efficacy of tDCS and PES to date has focussed on stroke populations. Nonetheless, several case reports, observational studies, and randomized controlled trials have suggested that tDCS may benefit patients with multiple sclerosis and Parkinson's disease [[25], [26], [27]], whereas PES may benefit patients with multiple sclerosis, traumatic brain injury, and other neurological conditions, with or without mechanical ventilation and tracheotomy [28,29].

Nonetheless, the generalizability of neurostimulation is limited by individual variability in treatment response [[30], [31], [32], [33]]. Such variability can be minimised by inducing “metaplasticity” [34], a homeostatic mechanism that regulates the nervous system's response to neuroplasticity-inducing stimuli [[35], [36], [37]], in the swallowing system [34]. Metaplasticity can keep modulations within a physiological dynamic range, i.e. neurons already in a state of high activation will have an increased threshold for further excitatory plasticity and vice versa. In humans, preconditioning the pharyngeal motor cortex with non-invasive brain stimulation (NIBS) can enhance its response to subsequent neurostimulation, improving the overall treatment outcomes [34]. Other studies targeting the primary motor cortex have shown that preconditioning with cathodal tDCS can switch the response to a subsequent session of cathodal tDCS from inhibition to facilitation [38] and potentiates the excitatory effects of subsequent 5Hz repetitive transcranial magnetic stimulation (rTMS) [39]. However, it is not yet known whether cross-modal (cortical-peripheral) stimulation, for example combined tDCS and PES, will induce metaplasticity.

Our study aimed to investigate PES preconditioned with tDCS on the cortical activation during swallowing and swallowing function in healthy adults. Magnetoencephalography (MEG) was used to detect and localize the power changes in cortical oscillatory activity during swallowing, as established in previous studies [18,20,40]. Changes in the synchrony of neuronal activity are reflected by event-related desynchronization (ERD) and event-related synchronization (ERS), which indicate cortical activation and inhibition, respectively [41].

## Materials and Methods

### Ethical approval

This study was approved by the local Ethics Committee of the Chamber of Physicians of Westfalen-Lippe (reference: 2023-142-f-S). Written informed consent was obtained before the commencement of study. All experimental procedures were conducted in accordance with the Declaration of Helsinki.

### Participants

Twenty-one healthy adults (8 males, 13 females; mean age = 27.0 ± 4.8 years) participated in this study. All participants fulfilled the safety criteria for tDCS [42]. They were free of any neurologic, psychiatric, swallowing or ear-nose-throat disorder, and did not take any medications affecting the central nervous system. They were right-handed as assessed by the Edinburgh Handedness Inventory (EHI) [43] (mean EHI score = 92.0; range = 73.3–100.0).

### Experimental outline

**Fig. 1:**
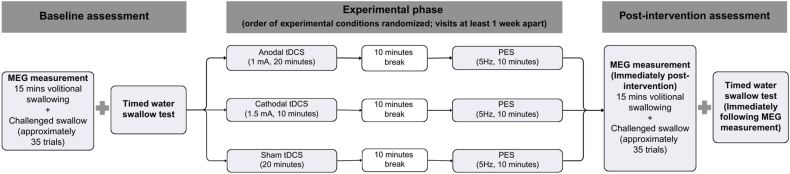
Flow diagram showing the study design. MEG: magnetoencephalography; PES: pharyngeal electrical stimulation; tDCS: transcranial direct current stimulation.

This randomized controlled trial employed a crossover design (Fig. 1). Each participant received three experimental conditions, including (i) anodal tDCS followed by PES (anodal tDCS + PES), (ii) cathodal tDCS followed by PES (cathodal tDCS + PES), and (iii) sham tDCS followed by PES (sham tDCS + PES), in randomized order on separate sessions with at least one week apart to avoid any carryover effects.

In each session, the cortical activation during swallowing was measured by MEG for 15 min and the swallowing function was assessed using the timed water swallow test (TWST) [44] before (baseline) and after (post-intervention) the intervention. Following baseline assessments, participants received the “preconditioning (anodal, cathodal or sham) tDCS”, followed by a 10-min break, and then PES. The time interval between preconditioning tDCS and the subsequent neuromodulation (PES) is critical for inducing metaplasticity [38]. Metaplasticity is optimally induced when the second stimulation occurs at the peak, or within the middle third, of the after-effects of the preconditioning stimulation [38,45]. The chosen 10-min interval was based on the study by Jefferson and colleagues [17], which showed that the anodal tDCS after-effects peaked between 0- and 15-min post-stimulation, while cathodal tDCS after-effects lasted approximately 30 min. A 10-min break following the preconditioning tDCS therefore falls within the peak period for anodal tDCS and the middle third of cathodal tDCS after-effects, while respecting the logistical constraints of our local facility. Personnel involved in data acquisition, preprocessing and analysis and participants were blinded towards the order and type of intervention.

### Experimental techniques

#### Transcranial direct current stimulation (tDCS)

TDCS was delivered by a battery-driven constant current stimulator (NeuroConn GmbH, Ilmenau, Germany) through a pair of conductive-rubber electrodes in two saline-soaked sponges. The stimulation target was right pharyngeal motor cortex, which was determined based on previous neurophysiological and MEG findings of cortical regions associated with swallowing [5,[46], [47], [48]], and right hemispheric tDCS being more effective than left hemispheric tDCS [18]. The active (anodal or cathodal) electrode (5 × 7 cm^2^) was positioned with the centre of electrode 3.5 cm lateral and 1 cm anterior to the vertex with its long axis parallel to the central sulcus [5,18,19]. The reference electrode (10 × 10 cm^2^) was placed over the contralateral supraorbital ridge. The large reference electrode was used to reduce local current density thereby diminishing its effect [49]. Anodal and cathodal tDCS was delivered at 1 mA for 20 min and at 1.5 mA for 10 min respectively, with a 30-s ramp-up and ramp-down of current at the beginning and end of stimulation [17]. Sham tDCS involved 30 s of current with electrodes left in place for 20 min, which evoked initial tingling sensation without sufficient cortical stimulation [50]. During tDCS, participants sucked on a lollipop and were offered still water to drink to maximize sensory input to cortical swallowing centres and to activate the swallowing network [18]. At the end of each session, participants rated the level of pain and discomfort on a 1 to 10 visual analogue scale (VAS) (1 = no discomfort, 10 = maximal pain) [51], guessed whether they received real or sham stimulation.

#### Pharyngeal electrical stimulation (PES)

PES was delivered using a 3.2 mm intraluminal catheter (Gaeltec, Isle of Skye, UK) with two bipolar platinum ring electrodes. The catheter was inserted transnasally with electrodes positioned in the pharynx. The catheter was connected to a constant current stimulator (Model DS7A) controlled by a trigger generator (Model DG2A, Digitimer Limited, Welwyn-Garden City, Herts, UK). The stimuli were delivered at the optimal parameters for enhancing pharyngeal cortical excitability (0.2 ms pulse duration, 5Hz, 280V, 10 min) [10]. The PES stimulation intensity was customized for each participant at 75 % of their maximum tolerated threshold, as established in a previous study [11].

#### Timed water swallow test (TWST)

Swallowing function was assessed by TWST. Each participant drank 150 ml of room temperature still water from a cup “as quickly as is comfortable” [44]. The duration from water touching the bottom lip to the throat resting after the final swallow was timed using a stopwatch. The number of swallows was counted by observing the upward-downward movements of the thyroid cartilage. The volume per swallow (ml), duration per swallow (s) and swallowing capacity (ml/s) were calculated for each participant.

#### Magnetoencephalography (MEG)

##### MEG system set-up

MEG data acquisition was performed as previously described [12,13,18,52]. The MEG system with a whole-head 275-channel SQUID sensor array (Omega 275, CTF Systems Inc.) installed within a magnetically shielded room measured the cortical activation during swallowing. Magnetic fields were recorded with a sample frequency of 600 Hz and filtered online using a 150Hz low-pass filter.

##### Electromyography (EMG)

The onset and duration of swallowing acts were identified by surface EMG recording with bipolar silver-chloride skin electrodes placed on the submental muscle groups. The electrodes were connected to a bipolar amplifier (DSQ 2017E EOG/EMG system, CTF Systems Inc., Canada) and co-registered with the MEG data. Trigger markers (see below) were co-registered parallel to MEG and EMG data for challenged swallow events.

##### Swallowing tasks

During MEG, participants sat upright looking at a black screen 80 cm away. Participants performed two swallow tasks: volitional swallow task and challenged swallow task (approximately 35 trials) as previously published [53]. To facilitate swallowing, room temperature still water was infused into the mouth via a 4.7 mm diameter flexible plastic tube attached to a fluid reservoir. The tube's tip was randomly placed in the either buccal cavity and taped to the skin at the mouth's corner. The infusion flow rate was approximately 12 ml/min. The “volitional swallow task” was performed during the first 15 min of MEG. Participants swallowed the infused water at their own pace without external cues. The “challenged swallow task” was adopted and modified from previous studies [18,54]. Participants swallowed within a target time interval upon a visual cue. During the task, the black screen displayed a green ring and a white solid circle expanding exocentrically from its centre over time. Participants were instructed to swallow when the white solid circle contacted the green ring, which lasted for 300 ms. Visual feedback on the accuracy of attempt (hit, early, late or miss) was given to the participants based on their EMG activity. “Presentation” was used as software application (NeuroBehavioral Systems, Inc., Berkeley, USA) for precise visual stimulus and feedback delivery on the screen.

The EMG characteristics, including swallow duration, EMG peak-to-peak amplitude, and EMG power (root mean square [RMS] value) [12,53], during both volitional and challenged swallow tasks, and the percentage of “hits” during challenged swallow task were analysed. Moreover, the swallow count during volitional swallow task and head movement during MEG measurement were recorded to ensure comparable performance across conditions.

### Data analysis

#### Behavioural data

Behavioural data were analysed using SPSS Statistics 27.0 (IBM Corporation, USA). Characteristics of PES stimulation parameters, including perceptual threshold (mA), maximum tolerated threshold (mA) and optimal stimulation intensity (mA), and VAS for perceived pain and discomfort, were compared across conditions using Friedman test. Differences in the participants' guesses of real versus sham tDCS were compared across conditions using Chi-square test. Two-way repeated measure ANOVAs were used to evaluate the effects of intervention and time on the performance in the TWST and challenged swallow task, and behavioural data during MEG measurements. Mauchly's test was used to evaluate the sphericity assumption on the data, and Greenhouse-Geisser correction was applied in case of violation. If significant effects of intervention or interactions were identified, the changes in the dependent variables from baseline were compared across interventions using paired *t*-tests with Bonferroni adjustment for multiple comparisons. Significance was set at *p* < 0.05.

#### MEG data

MEG data preprocessing and analysis was performed according to a previously established pipeline by our group, which has been used in manyfold previous MEG studies on swallowing [12,13,18]. In brief, MEG data processing and statistical analysis were performed using custom-made Matlab (MathWorks Inc., USA) scripts based on FieldTrip (http://www.ru.nl/fcdonders/fieldtrip) [55]. MEG data were filtered within five frequency bands, including theta (4–8 Hz), alpha (8–13 Hz), beta (13–30 Hz), low gamma (30–60 Hz) and high gamma (60–80 Hz). For event-related MEG data analysis the co-registered EMG signal was used to identify the onset and duration of swallowing. Source localization of each participant's ERD during swallowing was performed separately in all frequency bands using a linear constrained minimum variance (LCMV) beamformer technique and a volume conduction model from a canonical T1-weighted magnetic resonance imaging (MRI) in FieldTrip. Afterwards volumetric source estimates were spatially normalized to a template brain (T1, Montreal Neurological Institute, Canada). Grandaverages of normalized source activation maps were computed across participants for baseline and post-intervention data in all experimental conditions for each frequency band. Grandaverages were then interpolated onto a template MRI for localization and visualization of active regions using the WFU-PickAtlas tool (http://fmri.wfubmc.edu/). A cluster-based nonparametric randomization approach built into FieldTrip was applied to identify source locations with significant (*p* < 0.05) changes post-intervention [56]. To avoid source reconstruction artefact from tongue movement during swallowing, statistical analysis was restricted to predefined regions of interest (ROIs) comprising the frontal and parietal lobes and the insula, which are consistently associated with swallowing in previous neuroimaging studies and can be reliably localized with MEG [12,13,18,57].

## Results

No adverse effects were reported from the participants across conditions. There were no significant differences in characteristics of PES stimulation parameters among the three experimental conditions (Table 1). The level of discomfort during tDCS was minimal (VAS = 1.50 ± 0.33), and participants could not differentiate between real or sham stimulation (*p* = 0.145).

**Table 1:** Characteristics of PES stimulation parameters. Data are presented in mean ± standard deviation.

	Sham tDCS + PES	Anodal tDCS + PES	Cathodal tDCS + PES	*p* value
*PES*				
Perceptual threshold (mA)	2.57 ± 1.43	2.62 ± 1.73	2.79 ± 2.12	0.953
Maximum tolerated threshold (mA)	6.82 ± 4.07	5.95 ± 3.13	6.86 ± 4.56	0.651
Calculated optimal stimulation intensity (mA)	5.65 ± 3.36	5.11 ± 2.72	5.84 ± 3.86	0.774

PES: pharyngeal electrical stimulation; tDCS: transcranial direct current stimulation.

### Timed water swallow test (TWST)

There were significant effects of intervention (*F*_2,40_ = 3.495, *p* = 0.040) and time (*F*_1,20_ = 7.775, *p* = 0.011) on volume per swallow (Table 2). Post hoc analysis showed that the volume per swallow was significantly greater after intervention than baseline (*p* = 0.011), but there were no significant differences across interventions. There was no significant interaction (intervention x time) on volume per swallow (*F*_2,40_ = 0.395, *p* = 0.676).

**Table 2:** Results of timed water swallow test (TWST) performance at baseline and post-intervention. Data are expressed in mean ± standard deviation.

	Baseline	Post-intervention
*Volume per swallow (ml)*		
Sham tDCS + PES	38.57 ± 12.61	40.71 ± 11.78
Anodal tDCS + PES	37.14 ± 10.88	38.33 ± 12.20
Cathodal tDCS + PES	39.05 ± 11.50	41.90 ± 11.37
*Duration per swallow (s)*		
Sham tDCS + PES	1.12 ± 0.16	1.17 ± 0.34
Anodal tDCS + PES	1.13 ± 0.25	1.18 ± 0.28
Cathodal tDCS + PES	1.17 ± 0.34	1.19 ± 0.26
*Swallowing capacity (ml/s)*		
Sham tDCS + PES	34.89 ± 12.58	34.30 ± 10.69
Anodal tDCS + PES	33.85 ± 11.93	33.83 ± 11.73
Cathodal tDCS + PES	35.31 ± 11.99	36.42 ± 12.82

PES: pharyngeal electrical stimulation; tDCS: transcranial direct current stimulation.

No significant effects of intervention (*F*_2,40_ = 0.386, *p* = 0.682), time (*F*_1,20_ = 1.414, *p* = 0.248), and no interaction (*F*_2,40_ = 0.065, *p* = 0.938) were found for duration per swallow. For swallowing capacity, the effects of intervention (*F*_2,40_ = 1.630, *p* = 0.209), time (*F*_1,20_ = 0.029, *p* = 0.865) and their interaction (*F*_2,40_ = 0.512, *p* = 0.603) were nonsignificant.

### Behavioural data during MEG measurements

As intended, task execution during MEG measurements was within quality standards (reasonable number of swallows, head movement below 0.5 cm) and similar for all measurements (Table 3). The swallow count during volitional swallow task and head movement during MEG measurements were comparable across conditions. No significant effects of intervention (*F*_2,40_ = 1.282, *p* = 0.289), time (*F*_1,20_ = 0.049, *p* = 0.827) or interaction (*F*_2,40_ = 1.602, *p* = 0.214) on swallow count during volitional swallow task were found. Similarly, there were no significant effects of intervention or time, or interaction on head movement during volitional (intervention: *F*_2,36_ = 1.316, *p* = 0.281; time: *F*_1,18_ = 1.010, *p* = 0.328; interaction: *F*_2,36_ = 0.787, *p* = 0.463) or challenged (intervention: *F*_2,36_ = 1.025, *p* = 0.369; time: *F*_1,18_ = 0.006, *p* = 0.938; interaction: *F*_2,36_ = 1.076, *p* = 0.352) swallow tasks.

**Table 3:** MEG task performance data. Data are presented in mean ± standard deviation.

	Volitional swallow task		Challenged swallow task	
	Baseline	Post-intervention	Baseline	Post-intervention
*Head movement (cm)*				
Sham tDCS + PES	0.46 ± 0.36	0.54 ± 0.38	0.35 ± 0.23	0.33 ± 0.21
Anodal tDCS + PES	0.53 ± 0.48	0.44 ± 0.22	0.34 ± 0.26	0.39 ± 0.30
Cathodal tDCS + PES	0.66 ± 0.62	0.57 ± 0.44	0.41 ± 0.32	0.38 ± 0.29
*Swallow count during volitional swallow task*				
Sham tDCS + PES	85 ± 37	83 ± 33		
Anodal tDCS + PES	73 ± 24	78 ± 25		
Cathodal tDCS + PES	81 ± 41	76 ± 35		
*Percentage of hits during challenged swallow task (%)*				
Sham tDCS + PES			76.9 ± 11.0	73.2 ± 13.4
Anodal tDCS + PES			73.2 ± 13.4	75.0 ± 15.1
Cathodal tDCS + PES			70.7 ± 17.3	80.5 ± 16.2
*Swallow duration based on EMG activity (s)*				
Sham tDCS + PES	1.28 ± 0.30	1.39 ± 0.40	1.25 ± 0.25	1.39 ± 0.49
Anodal tDCS + PES	1.23 ± 0.25	1.33 ± 0.35	1.26 ± 0.27	1.37 ± 0.40
Cathodal tDCS + PES	1.32 ± 0.37	1.46 ± 0.44	1.27 ± 0.27	1.33 ± 0.29
*EMG power (μV)*				
Sham tDCS + PES	87.21 ± 101.79	60.39 ± 73.29	70.48 ± 45.72	60.21 ± 38.76
Anodal tDCS + PES	56.99 ± 25.94	45.88 ± 21.27	66.03 ± 45.28	73.34 ± 91.27
Cathodal tDCS + PES	94.78 ± 143.80	67.10 ± 89.12	130.43 ± 240.92	68.10 ± 67.96
*EMG peak-to-peak amplitude (μV)*				
Sham tDCS + PES	291.74 ± 156.59	272.81 ± 126.91	277.82 ± 96.77	296.86 ± 99.56
Anodal tDCS + PES	242.37 ± 76.07	263.31 ± 80.52	287.19 ± 120.97	335.98 ± 183.24
Cathodal tDCS + PES	344.78 ± 340.20	320.86 ± 284.81	412.43 ± 429.93	346.66 ± 256.20

EMG: electromyography; MEG: magnetoencephalography; PES: pharyngeal electrical stimulation; tDCS: transcranial direct current stimulation.

#### Volitional swallow task

There was a significant effect of time on swallow duration (*F*_1,20_ = 12.382, *p* = 0.002), but no significant effects of intervention (*F*_2,36_ = 0.719, *p* = 0.493) and no interaction (*F*_2,40_ = 0.169, *p* = 0.845). Similarly, there was a significant main effect of time on EMG power (*F*_1,19_ = 9.185, *p* = 0.007), but no significant effect of intervention (*F*_2,38_ = 1.447, *p* = 0.246) and no interaction (*F*_2,38_ = 2.088, *p* = 0.157).

There was a significant (time x intervention) interaction (*F*_2,38_ = 3.501, *p* = 0.040) on EMG peak-to-peak amplitude. However, post hoc paired *t*-tests revealed no significant differences in the changes in amplitude between sham tDCS + PES and anodal tDCS + PES (*p* = 0.029; nonsignificant after Bonferroni correction) or cathodal tDCS + PES (*p* = 0.903). There were no significant main effects of intervention *(F*_2, 38_ = 1.323, *p* = 0.270) or time (*F*_1,19_ = 0.287, *p* = 0.598).

#### Challenged swallow task

There was a significant main effect of time on swallow duration (*F*_1,20_ = 8.821, *p* = 0.008), but no significant effects of intervention (*F*_2,40_ = 0.034, *p* = 0.966), nor interaction (*F*_2,40_ = 0.294 *p* = 0.664). For EMG power, no significant main effects of intervention (*F*_2,40_ = 0.908, *p* = 0.371) or time (*F*_1,20_ = 2.268, *p* = 0.148), and no interaction (*F*_2,40_ = 2.304, *p* = 0.140) were found. Similarly, for EMG peak-to-peak amplitude, no significant main effects of intervention (*F*_2,40_ = 1.186, *p* = 0.300) or time (*F*_1,20_ = 0.001, *p* = 0.974), and no interaction (*F*_2,40_ = 2.997, *p* = 0.086) were found. No significant main effects of intervention (*F*_2,40_ = 0.485, *p* = 0.619), time (*F*_1,20_ = 3.805, *p* = 0.065) or interaction (*F*_2,40_ = 2.427, *p* = 0.101) were found for the percentage of hits during challenged swallow task.

### Cortical swallowing activation

#### Baseline cortical activation

**Fig. 2:**
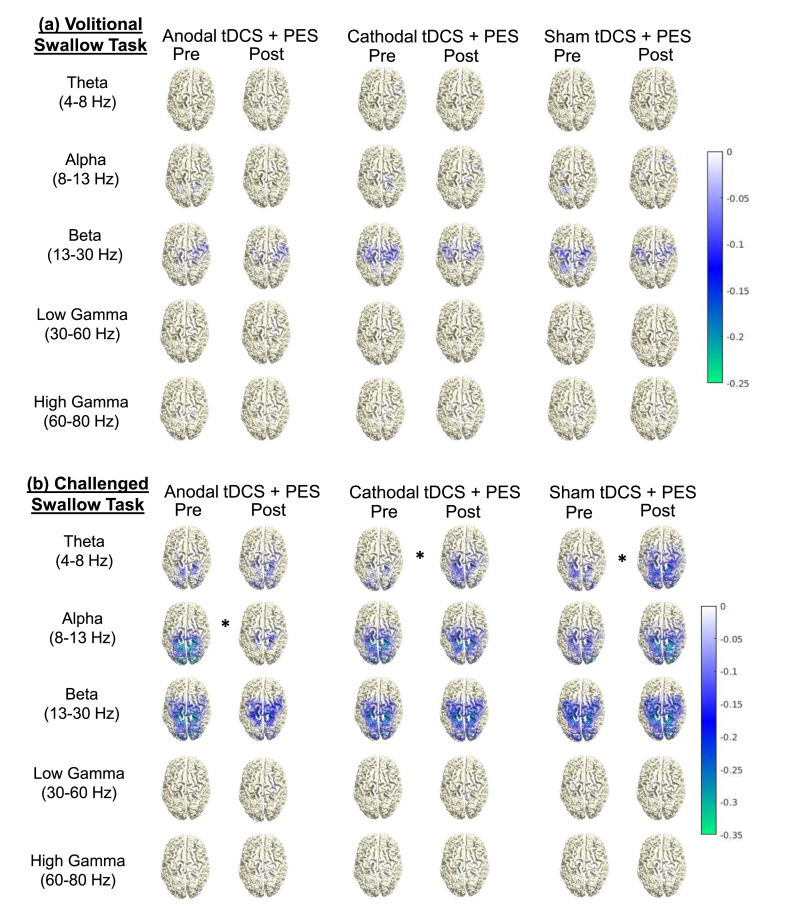
Source distribution of group averaged swallow-related cortical activation at all frequency bands for the (**a**) volitional swallow task and (**b**) challenged swallow task. Data are displayed for all three experimental conditions (anodal tDCS + PES, cathodal tDCS + PES and sham tDCS + PES) at baseline and post-intervention. Negative values denote event-related desynchronization (ERD) of oscillatory activity relative to the resting stage. Significant changes in ERD from baseline to post-intervention are highlighted with asterisks (∗).

Source distributions of group-averaged ERD during volitional and challenged swallow tasks are shown in Fig. 2. Swallow-related ERD was observed from theta to low gamma frequency band, with most prominent activation in the alpha and beta frequency range. No swallow-related ERD was detected in the high gamma frequency band. Swallow-associated activation was observed in both hemispheres and predominantly localized in the pericentral cortex, corresponding to primary and secondary sensorimotor areas. While swallow-related ERD were detected in both hemispheres, stronger activation was found in the right hemisphere. For the visually cued challenged swallow task, peak ERD was naturally identified in the primary and secondary visual cortex (BA 18, 19), as visual input was intense and time-locked to the analysis interval. To exclude visual cortex activation from the statistical comparison, the occipital lobe was not within the ROI.

#### Intervention effects

**Fig. 3:**
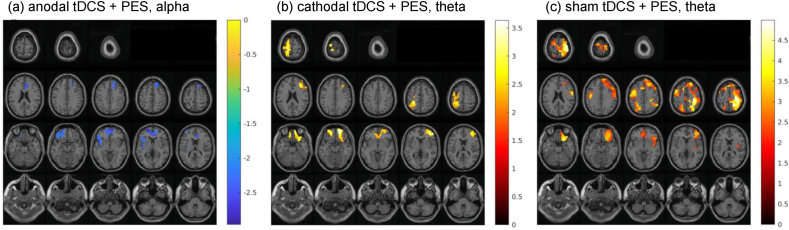
Diagrams showing the anatomical localization and color-coded intensity of significant group averaged changes in event-related desynchronization (ERD) of oscillatory brain activity during challenged swallow task after (**a**) anodal tDCS + PES, (**b**) cathodal tDCS + PES, and (**c**) sham tDCS + PES. ERD are shown for (**a**) alpha (8–13 Hz) and (**b, c**) theta (4–8 Hz) frequency ranges. Negative values indicate significant (*p* < 0.05) attenuation of ERD, while positive values indicate significant (*p* < 0.05) increase in ERD. L: left, R: right.

There were no significant changes in swallow-associated ERD during the simple volitional swallow task following any of the three interventions in our collective of young, healthy participants. By contrast, significant changes in swallow-related ERD were observed during the more demanding challenged swallow task in the theta and alpha frequency ranges after intervention (Fig. 3). Anodal tDCS + PES induced a significant attenuation in task-related ERD in the alpha frequency (*p* = 0.037). The attenuation in ERD was observed in both hemispheres, with the greatest reduction (*t*-value: 3.53) located in the left anterior prefrontal cortex. Other regions with significant attenuation included the left insula (BA 13), bilateral anterior cingulate cortex (BA 24, 32), frontal gyrus (BA 8, 10, 45–47), premotor and supplementary motor cortices (BA 6), and orbitofrontal cortex (BA 11).

Following cathodal tDCS + PES, a significant power increase of ERD was found in the theta frequency range (*p* = 0.042). The increase was observed bilaterally, with the most significant increase (*t*-value: 4.04) localized in the left primary motor cortex (BA 4). Prominent increase in ERD was also observed in regions including the left primary sensory cortex (BA 3) and sensory association cortices (BA 5, 7), right dorsolateral prefrontal cortex (DLPFC; BA 46), inferior frontal gyrus (BA47) and insular cortex (BA 16), and bilateral frontal gyrus (BA 8–10, 45–47), premotor and supplementary motor cortices (BA 6), orbitofrontal cortex (BA 11), supramarginal gyrus of the IPL (BA 40) and anterior cingulate cortex (BA 24, 32).

Similarly, significant increase in task-related ERD was found in the theta frequency range following sham tDCS + PES (*p* < 0.001). The increase in ERD was most significant (*t*-value: 5.75) in the right primary motor cortex (BA 4). Significant increases were also observed in regions including the left primary motor cortex (BA 4), right insular cortex (BA13), bilateral premotor and supplementary motor cortices (BA 6), frontal gyrus (BA 8–10, 45, 47), dorsolateral prefrontal cortex (DLPFC; BA 46), superior parietal gyrus corresponding to sensory association cortices (BA 5, 7), primary sensory cortex (BA 3, 2), supramarginal gyrus (BA40), angular gyrus (BA 39), orbitofrontal cortex (BA11), and anterior cingulate cortex (BA 32).

## Discussion

To our knowledge, this study provides the first evidence of cross-modal (cortical preconditioning followed by peripheral neurostimulation) metaplasticity in the human swallowing system. Although metaplasticity induced by sequential cortical stimulation (preconditioning tDCS followed by rTMS) is well established in the hand motor system [39,58], it has not been demonstrated in the swallowing system. Combined tDCS and neuromuscular electrical stimulation (NMES) protocols have shown preliminary benefits for post-stroke dysphagia. However, these studies did not investigate the polarity-dependent preconditioning effects or the neurophysiological mechanisms leading to the observed improvement. We investigated the changes in swallow-related oscillatory network and cortical activation patterns induced by PES preconditioned with three different protocols: anodal tDCS, cathodal tDCS, and sham tDCS. We found that PES alone, i.e. combined with sham tDCS, and preconditioning the pharyngeal motor cortex with cathodal tDCS prior to PES both lead to an increase of swallow-related event-related desynchronization (ERD) in the theta frequency band (4–8Hz), whereas anodal tDCS preconditioning had an opposite effect and attenuated swallow-related ERD in the alpha frequency band (8–13Hz). There were no significant changes in swallowing behaviour following intervention in young healthy volunteers with functional swallowing. These results provide novel insights into the potential of cross-modal neurostimulation in inducing metaplasticity in the human swallowing system.

### Cortical activation during swallowing

Baseline activation of cortical regions during swallowing was consistent with previous functional neuroimaging studies [40,47,[59], [60], [61]]. Swallow-related ERD was observed primarily in the primary sensorimotor cortex of both hemispheres, albeit asymmetrical, a finding consistent with previous studies [5,40,47]. Apart from the primary sensorimotor regions, activation was also observed in secondary sensorimotor regions. These areas may play a role in processing and integration of sensorimotor information during swallowing tasks [62]. In accordance with previous MEG studies, swallow-related ERD was most prominent in the alpha and beta frequency bands [12,18,61].

### Intervention effects on swallow-related oscillatory network and swallowing behaviour

Consistent with previous studies [12,18], neurostimulation-induced changes in ERD were identified in the stimulated pharyngeal motor cortex and a broad cortical network in both hemispheres, involving regions responsible for gustatory information processing, sensorimotor integration, and movement planning [1,59]. These changes were found primarily in the alpha and theta frequency bands. Oscillations in the alpha frequency band, which are associated with somatosensory integration and attention [63], attenuated following anodal tDCS-preconditioned PES, suggesting reduced allocation of attentional resources or effort [41,63]. On the other hand, oscillations in the theta frequency band, which are associated with hippocampus electrophysiological activity, spatial exploration, locomotion [[64], [65], [66]], and synaptic plasticity [67,68], increased after cathodal tDCS-preconditioned PES, suggesting enhanced motor processing and synaptic plasticity. Moreover, ERD changes were observed only during the challenged swallow task, which is an externally-cued task, but not volitional swallow task, likely due to its higher cognitive and attentional demand for stimulus processing and motor planning [18,63].

Interestingly, the direction of effects of preconditioned PES appeared to depend on the polarity of the preconditioning tDCS. Anodal tDCS have excitatory effects in the pharyngeal motor cortex [17] and swallowing neural network [18]. However, subsequent PES delivered to the aroused swallowing network resulted in downregulation with attenuation of swallow-related ERD in our study. This may suggest reduced somatosensory processing and sensorimotor integration in the swallowing network, similar to the effects observed after pharyngeal anaesthesia [57], and in senior individuals with cognitive decline during dual-task swallowing paradigms [52]. Such unexpected discrepancy may be explained by the metaplasticity [[34], [35], [36], [37]]. In our study, anodal tDCS excited the pharyngeal motor cortex and elevated the excitability threshold, making the pharyngeal motor cortex hypoexcitable. When excitatory PES was applied afterwards, metaplasticity mechanisms were activated such that the overall activation of cortical swallowing neural network was attenuated. This is in line with previous studies which showed that consecutive excitatory stimulation on motor cortex led to reduced overall neurostimulation effects [38,39].

By contrast, preconditioning with cathodal tDCS increased swallow-related ERD. It is plausible that the pharyngeal motor cortex was made hyperexcitable by cathodal tDCS [17]. Therefore, the response to the subsequent excitatory PES was enhanced, resulting in an overall increase in cortical activation of swallowing neural network. This finding is consistent with a previous metaplasticity study using repetitive transcranial magnetic stimulation (rTMS), in which inhibitory preconditioning of the pharyngeal motor cortex enhanced the effects of the subsequent excitatory rTMS [34]. Although we had previously reported that anodal tDCS applied alone can enhance swallow-related cortical activation [18], a direct quantitative comparison between anodal tDCS and cathodal tDCS + PES condition between trials is not possible. Nonetheless, both anodal tDCS alone and the current cathodal tDCS + PES protocol can lead to bilateral enhancement in swallowing-network excitability that extend beyond the stimulated hemisphere, suggesting that either approach can induce widespread neuroplasticity in the swallowing motor system. Future studies with head-to-head comparisons are needed to evaluate the relative efficacy of these approaches. Finally, the changes following sham tDCS preconditioning reflected the effects of PES alone, which increased the cortical activation of swallowing neural network, consistent with previous findings [9,10,12]. Although our previous MEG study suggested PES reduced swallow-related ERD in the alpha and beta frequency ranges [12], the task in which they found such attenuation was different from the challenged swallow task we used in our study. Therefore, the results are not directly comparable.

The intervention-induced changes in cortical activation were not associated with any behavioural changes, suggesting a potential ceiling effect. The target time window for swallowing during this task was 300 ms, as opposed to 150 ms used in previous studies [18,53]. As such, the average percentage of hits (over 76 %) in our cohort was much higher than reported in previous studies (40–60 %) [18,53]. This indicated that a more challenging task may be needed to reveal the differences in swallowing behaviour induced by the preconditioned protocols in young healthy subjects.

### Clinical implications and future directions

Our findings offer a new perspective on clinical application of cross-modal neurostimulation in dysphagia management such that existing dysphagia treatment effects may be optimised by preconditioning with tDCS. A common argument against the clinical routine use of neurostimulation is the considerable inter-individual variability in treatment response, which can limit overall effect sizes. This variability may, in part, be attributed to differences in patients’ baseline cortical activation states, which can make them more or less receptive to the intervention. Such variability can be reduced by leveraging the principles of metaplasticity—that is, by preconditioning the brain to establish a “baseline” state in which it is optimally primed to respond to subsequent PES. The present study was designed as a basic proof-of-principle investigation of these mechanisms in healthy volunteers. Although we were unable to conduct a direct statistical comparison of the effect sizes of the different preconditioning conditions prior to PES, our findings nevertheless suggest that cathodal tDCS effectively prepares the brain to respond to later PES, whereas anodal tDCS does not. These polarity-dependent preconditioning effects provide valuable insights for the development of future cross-modal (cortical–peripheral) neurostimulation protocols intended for clinical trials. Further studies may also explore the application of tDCS preconditioning in other peripheral stimulation approaches, such as carbonation or cold temperature [69,70], which have shown potential in inducing neuroplasticity changes in the swallowing system.

To our knowledge, no studies have explored cross-modal preconditioning in patients with post-stroke dysphagia. However, preconditioned NIBS protocols have been investigated in the primary motor cortex of stroke patients with upper-limb paresis [71,72]. For example, Cassidy et al. (2010) showed that preconditioning the motor cortex with 6Hz rTMS prior to 1Hz rTMS induced metaplasticity in the ipsilesional hemisphere, as evidenced by greater reduction in cortical silent period than 1Hz rTMS alone [71]. However, the preconditioned rTMS protocol did not consistently improve all neurophysiological measures or hand function, which could be attributed to heterogeneity in stroke characteristics. Similarly, a case report of 2 stroke patients showed that 6Hz rTMS preconditioning prior to 1Hz rTMS yielded mixed behavioural outcomes, which may be due to the differences in the extent and location of stroke lesion [72]. A recent randomized controlled trial showed that preconditioning intermittent theta burst stimulation (iTBS) before robot-assisted motor training did not yield additional benefits in chronic stroke patients compared to without preconditioning [73]. However, subgroup analysis revealed greater gains in patients with better upper limb function than those with worse function. Therefore, although preconditioned neurostimulation shows therapeutic promise in stroke, its efficacy may be dependent on individual stroke characteristics and residual functions. Further clinical studies on the biochemical effects of cross-modal neurostimulation are warranted to elucidate its mechanisms and its therapeutic potential in patients with neurogenic dysphagia.

### Limitations

The study cohort consisted of mainly young adults, whose neuroplasticity efficiency and mechanisms are known to be different from the elderly [[74], [75], [76], [77]]. With advancing age, the hemispheric control of swallowing become increasingly bilateral, which is believed to compensate for the age-related decline in swallowing function [78]. Compared with younger adults, older adults showed greater activation of somatosensory cortex, prefrontal cortex and middle temporal gyrus during swallowing, potentially implying compensatory recruitment of neural network or increased need for resources required for safe swallowing [48,79]. Moreover, the mechanisms of neuroplasticity, which is critical for recovery after brain damage such as stroke, become less efficient with age [76]. With regards to brain response to neurostimulation or preconditioned neurostimulation protocols, studies have demonstrated different responses between younger and older adults. Opie et al. (2017) found that preconditioning of the primary motor cortex with iTBS induced metaplasticity in younger but not in older adults [80]. Park et al. (2017) also showed that 5Hz rTMS did not alter the cortical activation during swallowing in older adults [77]. These findings suggest age-dependent differences in neuroplasticity mechanisms. Further studies that compare the responses between younger and older adults are needed to clarify the therapeutic value of preconditioned protocols in the older population who are at high risk of neurological disorders, including stroke.

Moreover, the potential ceiling effect of the challenged swallow task may have masked the true intervention effects on swallowing performance. A more challenging task, for example a dual task paradigm [52], may be applied in future studies. Moreover, the TWST was used as one of the primary outcome measures. Although TWST provides quantifiable data on swallowing function, this test has limited sensitivity for detecting subclinical dysphagia or silent aspiration. Future studies in patients should incorporate instrumental assessments such as videofluoroscopy (VFSS) or fiberoptic endoscopic evaluation of swallowing (FEES) to provide more objective and comprehensive evaluation of changes in swallowing physiology.

Due to the technical requirements of an MEG measurement, especially minimization of movement artifacts, water was administered through an intraoral tube to facilitate swallowing, which may deviate from natural drinking. However, this approach has been used successfully in numerous previous MEG swallowing studies and the observed cortical activation patterns were consistent [12,18]. Moreover, we intended to measure a potential intervention effect on swallowing network and function by pre-post comparison. It was not our primary goal to disentangle the details of swallowing neurophysiology per se.

Regarding the comparisons of effects across conditions, due to methodological constraints, a direct head-to-head statistical comparison between cathodal tDCS + PES vs sham tDCS + PES was not pre-planned, as the primary aim of the study was to explore the polarity-dependent effects of tDCS preconditioning on PES-induced neuroplasticity. Further analyses will be needed to investigate whether cathodal tDCS + PES lead to more superior excitatory effects than without preconditioning. Finally, since MEG is most sensitive to changes in the cerebral cortex, any intervention-induced changes in the subcortical structures or brainstem may have gone undetected. Further investigations using complementary imaging techniques may provide better understanding of the differential effects on different levels of the swallowing neural system.

In summary, this study was the first to investigate the effects of cross-modal neurostimulation on the cortical activation of swallowing neural network and swallowing function. We found that PES preconditioned by cathodal tDCS increased whereas anodal preconditioned PES reduced swallow-related cortical activation. These findings suggested that potentially therapeutically beneficial metaplasticity mechanisms can be induced by preconditioning the pharyngeal motor cortex with cathodal tDCS prior to PES. Future studies may focus on its clinical application in patients with neurogenic dysphagia.

## CRediT authorship contribution statement

**Ivy Cheng**: Funding acquisition, Conceptualization, Methodology, Validation, Formal analysis, Investigation, Data Curation, Writing – original draft, Visualization, Project administration. **Anne Jung**: Methodology, Validation, Formal analysis, Investigation, Data Curation, Writing – original draft, Visualization, Project administration, Writing - Review & Editing. **Bendix Labeit**: Conceptualization, Methodology, Resources, Validation, Project administration, Writing - Review & Editing. **Rainer Dziewas**: Conceptualization, Methodology, Resources, Validation, Project administration, Writing - Review & Editing. **Andreas Wollbrink**: Methodology, Validation, Formal analysis, Data Curation, Writing – Review & Editing, Resources. **Joachim Gross**: Methodology, Resources, Writing – Review & Editing, Supervision. **Paul Muhle**: Methodology, Validation, Formal analysis, Investigation, Data Curation, Writing – Review & Editing, Visualization, Project administration, Supervision. **Sonja Suntrup-Krueger**: Funding acquisition, Conceptualization, Methodology, Validation, Formal analysis, Investigation, Data Curation, Resources, Writing – original draft, Writing – Review & Editing, Visualization, Project administration, Supervision.

## Declaration of competing interest

The authors declare the following financial interests/personal relationships which may be considered as potential competing interests: Ivy Cheng reports a relationship with University of Münster that includes: funding grants. Sonja Suntrup-Krueger reports a relationship with Else Kroner-Fresenius Foundation that includes: funding grants. Sonja Suntrup-Krueger reports a relationship with Phagenesis Ltd that includes: speaking and lecture fees. Paul Muhle reports a relationship with Phagenesis Ltd that includes: speaking and lecture fees. Bendix Labeit reports a relationship with German Research Foundation that includes: funding grants. Bendix Labeit reports a relationship with University of Münster that includes: funding grants. Bendix Labeit reports a relationship with German Society for Geriatrics that includes: funding grants. Bendix Labeit reports a relationship with Rolf und Hubertine Schiffbauer Foundation that includes: funding grants. Bendix Labeit reports a relationship with German Society for Clinical Neurophysiology and Functional Imaging that includes: travel reimbursement. Bendix Labeit reports a relationship with European Stroke Organisation that includes: travel reimbursement. Bendix Labeit reports a relationship with German Society for Neurology that includes: travel reimbursement. Bendix Labeit reports a relationship with European Society for Swallowing Disorders that includes: travel reimbursement. Bendix Labeit reports a relationship with United European Gastroenterology that includes: travel reimbursement. Bendix Labeit reports a relationship with German Society for Nutritional Medicine eV that includes: travel reimbursement. Bendix Labeit reports a relationship with German Society for Internal Medicine eV that includes: travel reimbursement. Bendix Labeit reports a relationship with University of Zurich that includes: travel reimbursement. Bendix Labeit reports a relationship with German Society for Muscular Diseases that includes: speaking and lecture fees. Bendix Labeit reports a relationship with Phagenesis Ltd that includes: speaking and lecture fees. If there are other authors, they declare that they have no known competing financial interests or personal relationships that could have appeared to influence the work reported in this paper.

## References

[1] Cheng I., Takahashi K., Miller A.J., Hamdy S. (2022). Cerebral control of swallowing: an update on neurobehavioral evidence. J Neurol Sci.

[2] Martino R., Foley N., Bhogal S., Diamant N., Speechley M., Teasell R. (2005). Dysphagia after stroke: incidence, diagnosis, and pulmonary complications. Stroke.

[3] Ekberg O., Hamdy S., Woisard V., Wuttge–Hannig A., Ortega P. (2002). Social and psychological burden of dysphagia: its impact on diagnosis and treatment. Dysphagia.

[4] Marin S., Serra-Prat M., Ortega O., Audouard Fericgla M., Valls J., Palomera E. (2021). Healthcare costs of post-stroke oropharyngeal dysphagia and its complications: malnutrition and respiratory infections. Eur J Neurol.

[5] Hamdy S., Aziz Q., Rothwell J.C., Singh K.D., Barlow J., Hughes D.G. (1996). The cortical topography of human swallowing musculature in health and disease. Nat Med.

[6] Hamdy S., Aziz Q., Rothwell J.C., Power M., Singh K.D., Nicholson D.A. (1998). Recovery of swallowing after dysphagic stroke relates to functional reorganization in the intact motor cortex. Gastroenterology.

[7] Suntrup S., Teismann I., Bejer J., Suttrup I., Winkels M., Mehler D. (2013). Evidence for adaptive cortical changes in swallowing in Parkinson's disease. Brain.

[8] Dziewas R., Teismann I.K., Suntrup S., Schiffbauer H., Steinstraeter O., Warnecke T. (2009). Cortical compensation associated with dysphagia caused by selective degeneration of bulbar motor neurons. Hum Brain Mapp.

[9] Hamdy S., Rothwell J.C., Aziz Q., Singh K.D., Thompson D.G. (1998). Long-term reorganization of human motor cortex driven by short-term sensory stimulation. Nat Neurosci.

[10] Fraser C., Power M., Hamdy S., Rothwell J., Hobday D., Hollander I. (2002). Driving plasticity in human adult motor cortex is associated with improved motor function after brain injury. Neuron.

[11] Fraser C., Rothwell J., Power M., Hobson A., Thompson D., Hamdy S. (2003). Differential changes in human pharyngoesophageal motor excitability induced by swallowing, pharyngeal stimulation, and anesthesia. Am J Physiol Gastrointest Liver Physiol.

[12] Suntrup S., Teismann I., Wollbrink A., Winkels M., Warnecke T., Pantev C. (2015). Pharyngeal electrical stimulation can modulate swallowing in cortical processing and behavior—Magnetoencephalographic evidence. Neuroimage.

[13] Muhle P., Labeit B., Wollbrink A., Claus I., Warnecke T., Wolters C.H. (2021). Targeting the sensory feedback within the swallowing network—Reversing artificially induced pharyngolaryngeal hypesthesia by central and peripheral stimulation strategies. Hum Brain Mapp.

[14] Suntrup-Krueger S., Bittner S., Recker S., Meuth S.G., Warnecke T., Suttrup I. (2016). Electrical pharyngeal stimulation increases substance P level in saliva. Neuro Gastroenterol Motil.

[15] Nitsche M.A., Paulus W. (2000). Excitability changes induced in the human motor cortex by weak transcranial direct current stimulation. J Physiol.

[16] Nitsche M.A., Paulus W. (2001). Sustained excitability elevations induced by transcranial DC motor cortex stimulation in humans. Neurology.

[17] Jefferson S., Mistry S., Singh S., Rothwell J., Hamdy S. (2009). Characterizing the application of transcranial direct current stimulation in human pharyngeal motor cortex. Am J Physiol Gastrointest Liver Physiol.

[18] Suntrup S., Teismann I., Wollbrink A., Winkels M., Warnecke T., Floel A. (2013). Magnetoencephalographic evidence for the modulation of cortical swallowing processing by transcranial direct current stimulation. Neuroimage.

[19] Suntrup-Krueger S., Ringmaier C., Muhle P., Wollbrink A., Kemmling A., Hanning U. (2018). Randomized trial of transcranial direct current stimulation for poststroke dysphagia. Ann Neurol.

[20] Suntrup S., Marian T., Schroder J.B., Suttrup I., Muhle P., Oelenberg S. (2015). Electrical pharyngeal stimulation for dysphagia treatment in tracheotomized stroke patients: a randomized controlled trial. Intensive Care Med.

[21] Suntrup-Krueger S., Labeit B., von Itter J., Jung A., Claus I., Ahring S. (2025). Treating postextubation dysphagia after stroke with pharyngeal electrical stimulation–insights from a randomized controlled pilot trial. Neurotherapeutics.

[22] Dziewas R., Stellato R., van der Tweel I., Walther E., Werner C.J., Braun T. (2018). Pharyngeal electrical stimulation for early decannulation in tracheotomised patients with neurogenic dysphagia after stroke (PHAST-TRAC): a prospective, single-blinded, randomised trial. Lancet Neurol.

[23] Cheng I., Sasegbon A., Hamdy S. (2021). Effects of neurostimulation on poststroke Dysphagia: a synthesis of current evidence from randomized controlled trials. Neuromodulation.

[24] Suntrup-Krueger S., Labeit B., Marian T., Schröder J., Claus I., Ahring S. (2023). Pharyngeal electrical stimulation for postextubation dysphagia in acute stroke: a randomized controlled pilot trial. Crit Care.

[25] Cosentino G., Gargano R., Bonura G., Realmuto S., Tocco E., Ragonese P. (2018). Anodal tDCS of the swallowing motor cortex for treatment of dysphagia in multiple sclerosis: a pilot open-label study. Neurol Sci.

[26] Restivo D.A., Alfonsi E., Casabona A., Hamdy S., Tassorelli C., Panebianco M. (2019). A pilot study on the efficacy of transcranial direct current stimulation applied to the pharyngeal motor cortex for dysphagia associated with brainstem involvement in multiple sclerosis. Clin Neurophysiol.

[27] Dashtelei A.A., Nitsche M.A., Salehinejad M.A., Habibi A.H., Bakhtyiari J., Khatoonabadi A.R. (2024). Adjunctive transcranial direct current stimulation to improve swallowing functions in Parkinson's disease. EXCLI J.

[28] Restivo D.A., Casabona A., Centonze D., Marchese-Ragona R., Maimone D., Pavone A. (2013). Pharyngeal electrical stimulation for dysphagia associated with multiple sclerosis: a pilot study. Brain Stimul.

[29] Bath P.M., Woodhouse L.J., Suntrup-Krueger S., Likar R., Koestenberger M., Warusevitane A. (2020). Pharyngeal electrical stimulation for neurogenic dysphagia following stroke, traumatic brain injury or other causes: main results from the PHADER cohort study. eClinicalMedicine.

[30] Hordacre B., Goldsworthy M.R., Vallence A.M., Darvishi S., Moezzi B., Hamada M. (2017). Variability in neural excitability and plasticity induction in the human cortex: a brain stimulation study. Brain Stimul.

[31] Lopez-Alonso V., Cheeran B., Rio-Rodriguez D., Fernandez-Del-Olmo M. (2014). Inter-individual variability in response to non-invasive brain stimulation paradigms. Brain Stimul.

[32] Maeda F., Keenan J.P., Tormos J.M., Topka H., Pascual-Leone A. (2000). Modulation of corticospinal excitability by repetitive transcranial magnetic stimulation. Clin Neurophysiol.

[33] Raginis-Zborowska A., Cheng I., Pendleton N., Payton A., Ollier W., Michou E. (2019). Genetic influences on the variability of response to repetitive transcranial magnetic stimulation in human pharyngeal motor cortex. Neuro Gastroenterol Motil.

[34] Cheng I., Scarlett H., Zhang M., Hamdy S. (2020). Preconditioning human pharyngeal motor cortex enhances directional metaplasticity induced by repetitive transcranial magnetic stimulation. J Physiol.

[35] Abraham W.C., Bear M.F. (1996). Metaplasticity: the plasticity of synaptic plasticity. Trends Neurosci.

[36] Muller-Dahlhaus F., Ziemann U. (2015). Metaplasticity in human cortex. Neuroscientist.

[37] Bienenstock E.L., Cooper L.N., Munro P.W. (1982). Theory for the development of neuron selectivity: orientation specificity and binocular interaction in visual cortex. J Neurosci.

[38] Fricke K., Seeber A.A., Thirugnanasambandam N., Paulus W., Nitsche M.A., Rothwell J.C. (2011). Time course of the induction of homeostatic plasticity generated by repeated transcranial direct current stimulation of the human motor cortex. J Neurophysiol.

[39] Lang N., Siebner H.R., Ernst D., Nitsche M.A., Paulus W., Lemon R.N. (2004). Preconditioning with transcranial direct current stimulation sensitizes the motor cortex to rapid-rate transcranial magnetic stimulation and controls the direction of after-effects. Biol Psychiatry.

[40] Dziewas R., Sörös P., Ishii R., Chau W., Henningsen H., Ringelstein E.B. (2003). Neuroimaging evidence for cortical involvement in the preparation and in the act of swallowing. Neuroimage.

[41] Pfurtscheller G., Da Silva F.L. (1999). Event-related EEG/MEG synchronization and desynchronization: basic principles. Clin Neurophysiol.

[42] Antal A., Alekseichuk I., Bikson M., Brockmöller J., Brunoni A.R., Chen R. (2017). Low intensity transcranial electric stimulation: safety, ethical, legal regulatory and application guidelines. Clin Neurophysiol.

[43] Oldfield R.C. (1971). The assessment and analysis of handedness: the Edinburgh inventory. Neuropsychologia.

[44] Hughes T., Wiles C. (1996). Clinical measurement of swallowing in health and in neurogenic dysphagia. QJM: Int J Med.

[45] Hassanzahraee M., Zoghi M., Jaberzadeh S. (2018). How different priming stimulations affect the corticospinal excitability induced by noninvasive brain stimulation techniques: a systematic review and meta-analysis. Rev Neurosci.

[46] Teismann I.K., Steinsträter O., Warnecke T., Suntrup S., Ringelstein E.B., Pantev C. (2009). Tactile thermal oral stimulation increases the cortical representation of swallowing. BMC Neurosci.

[47] Teismann I.K., Dziewas R., Steinstraeter O., Pantev C. (2009). Time-dependent hemispheric shift of the cortical control of volitional swallowing. Hum Brain Mapp.

[48] Teismann I.K., Steinstraeter O., Schwindt W., Ringelstein E.B., Pantev C., Dziewas R. (2010). Age-related changes in cortical swallowing processing. Neurobiol Aging.

[49] Nitsche M.A., Doemkes S., Karakose T., Antal A., Liebetanz D., Lang N. (2007). Shaping the effects of transcranial direct current stimulation of the human motor cortex. J Neurophysiol.

[50] Gandiga P.C., Hummel F.C., Cohen L.G. (2006). Transcranial DC stimulation (tDCS): a tool for double-blind sham-controlled clinical studies in brain stimulation. Clin Neurophysiol.

[51] Huskisson E.C. (1974). Measurement of pain. Lancet.

[52] Suntrup-Krueger S., Muhle P., Slavik J., von Itter J., Wollbrink A., Wirth R. (2025). Cognitive decline limits compensatory resource allocation within the aged swallowing network. GeroScience.

[53] Suntrup-Krueger S., Muhle P., Kampe I., Egidi P., Ruck T., Lenze F. (2021). Effect of capsaicinoids on neurophysiological, biochemical, and mechanical parameters of swallowing function. Neurotherapeutics.

[54] Mistry S., Verin E., Singh S., Jefferson S., Rothwell J.C., Thompson D.G. (2007). Unilateral suppression of pharyngeal motor cortex to repetitive transcranial magnetic stimulation reveals functional asymmetry in the hemispheric projections to human swallowing. J Physiol.

[55] Oostenveld R., Fries P., Maris E., FieldTrip J.-M.S. (2011).

[56] Maris E., Oostenveld R. (2007). Nonparametric statistical testing of EEG-and MEG-data. J Neurosci Methods.

[57] Muhle P., Claus I., Marian T., Schröder J.B., Wollbrink A., Pantev C. (2019). Introducing a virtual lesion model of dysphagia resulting from pharyngeal sensory impairment. Neurosignals.

[58] Siebner H.R., Lang N., Rizzo V., Nitsche M.A., Paulus W., Lemon R.N. (2004). Preconditioning of low-frequency repetitive transcranial magnetic stimulation with transcranial direct current stimulation: evidence for homeostatic plasticity in the human motor cortex. J Neurosci.

[59] Hamdy S., Mikulis D.J., Crawley A., Xue S.W., Lau H., Henry S. (1999). Cortical activation during human volitional swallowing: an event-related fMRI study. Am J Physiol Gastrointest Liver Physiol.

[60] Hamdy S., Rothwell J.C., Brooks D.J., Bailey D., Aziz Q., Thompson D.G. (1999). Identification of the cerebral loci processing human swallowing with H-2 O-15 PET activation. J Neurophysiol.

[61] Furlong P.L., Hobson A., Aziz Q., Barnes G., Singh K.D., Hillebrand A. (2004). Dissociating the spatio-temporal characteristics of cortical neuronal activity associated with human volitional swallowing in the healthy adult brain. Neuroimage.

[62] Hamdy S., Mikulis D.J., Crawley A., Xue S., Lau H., Henry S. (1999). Cortical activation during human volitional swallowing: an event-related fMRI study. Am J Physiol.

[63] Neuper C., Pfurtscheller G. (2001). Event-related dynamics of cortical rhythms: frequency-specific features and functional correlates. Int J Psychophysiol.

[64] Buzsáki G. (2002). Theta oscillations in the hippocampus. Neuron.

[65] Tsanov M., Manahan-Vaughan D. (2009). Long-term plasticity is proportional to theta-activity. PLoS One.

[66] Kaplan R., Doeller C.F., Barnes G.R., Litvak V., Düzel E., Bandettini P.A. (2012). Movement-related theta rhythm in humans: coordinating self-directed hippocampal learning. PLoS Biol.

[67] Caplan J.B., Madsen J.R., Raghavachari S., Kahana M.J. (2001). Distinct patterns of brain oscillations underlie two basic parameters of human maze learning. J Neurophysiol.

[68] Lengyel M., Huhn Z., Érdi P. (2005). Computational theories on the function of theta oscillations. Biol Cybern.

[69] Michou E., Mastan A., Ahmed S., Mistry S., Hamdy S. (2012). Examining the role of carbonation and temperature on water swallowing performance: a swallowing reaction-time study. Chem Senses.

[70] Magara J., Michou E., Raginis-Zborowska A., Inoue M., Hamdy S. (2016). Exploring the effects of synchronous pharyngeal electrical stimulation with swallowing carbonated water on cortical excitability in the human pharyngeal motor system. Neuro Gastroenterol Motil.

[71] Cassidy J.M., Chu H., Anderson D.C., Krach L.E., Snow L., Kimberley T.J. (2015). A comparison of primed low-frequency repetitive transcranial magnetic stimulation treatments in chronic stroke. Brain Stimul.

[72] Carey J.R., Anderson D.C., Gillick B.T., Whitford M., Pascual-Leone A. (2010). 6-Hz primed low-frequency rTMS to contralesional M1 in two cases with middle cerebral artery stroke. Neurosci Lett.

[73] Zhang J.J., Bai Z., Fong K.N. (2022). Priming intermittent theta burst stimulation for hemiparetic upper limb after stroke: a randomized controlled trial. Stroke.

[74] Malandraki G.A., Perlman A.L., Karampinos D.C., Sutton B.P. (2011). Reduced somatosensory activations in swallowing with age. Hum Brain Mapp.

[75] Freitas C., Perez J., Knobel M., Tormos J.M., Oberman L., Eldaief M. (2011). Changes in cortical plasticity across the lifespan. Front Aging Neurosci.

[76] Todd G., Kimber T.E., Ridding M.C., Semmler J.G. (2010). Reduced motor cortex plasticity following inhibitory rTMS in older adults. Clin Neurophysiol.

[77] Park J.W., Sim G.J., Kim H.J., Yeo J.S., Hong H.J., Kwon B.S. (2017). Changes of cortical activation in swallowing following high frequency repetitive transcranial magnetic stimulation in older adults. Neuro Gastroenterol Motil.

[78] Malandraki G.A., Sutton B.P., Perlman A.L., Karampinos D.C. (2010). Age-related differences in laterality of cortical activations in swallowing. Dysphagia.

[79] Hyun Im Moon M., Jung Y., Choi S., Tae W., Pyun S.-B. (2016). Effect of age on cortical activation during swallowing: an fMRI study. J Kor Dysph Soci.

[80] Opie G.M., Vosnakis E., Ridding M.C., Ziemann U., Semmler J.G. (2017). Priming theta burst stimulation enhances motor cortex plasticity in young but not old adults. Brain Stimul.

